# The effect of future self-continuity on intertemporal decision making: a mediated moderating model

**DOI:** 10.3389/fpsyg.2024.1437065

**Published:** 2024-08-08

**Authors:** Ying Yang, Liangxiangwan Zhang, Weiguo Qu, Wei Fan

**Affiliations:** ^1^Department of Psychology, School of Education Science, Hunan Normal University, Changsha, China; ^2^Cognition and Human Behavior Key Laboratory of Hunan Province, Hunan Normal University, Changsha, China; ^3^Institute of Interdisciplinary Studies, Hunan Normal University, Changsha, China

**Keywords:** future self-continuity, self-concept clarity, future outcome consideration, intertemporal decision, mediated moderating model

## Abstract

Intertemporal decision making refers to the behavior of making decisions after weighing the costs and benefits of two or more outcomes at different time points. This study explores the moderating effect of self-concept clarity on the influence of future self-continuity on intertemporal decision-making and the mediating effect of future outcome consideration, aiming to establish a mediated moderating model. In Study 1, we recruited 370 participants via questionnaire to explore the relationship between future self-continuity and intertemporal decision-making, as well as the moderating effect of self-concept clarity. The results showed that: (1) Future self-continuity significantly negatively predicted the time discount rate of intertemporal decision-making. (2) Self-concept clarity significantly negatively moderated the relationship between future self-continuity and the time discount rate of intertemporal decision-making. In Study 2, we recruited 234 participants using an experimental method and divided them into high and low future self-continuity groups to explore the mediating effect of future outcome consideration and the moderating role of self-concept clarity in the influence of future self-continuity on intertemporal decision-making. The results indicated that: (1) Self-concept clarity significantly negatively moderated the impact of future self-continuity on future outcome consideration. (2) Future outcome consideration mediated the moderating effect of self-concept clarity on the influence of future self-continuity on intertemporal decision-making. The findings indicated that future self-continuity negatively impacted the time discount rate in intertemporal decision-making. Furthermore, self-concept clarity could indirectly regulate the effect of future self-continuity on intertemporal decision-making through future outcome consideration. These two studies contribute to a better understanding of intertemporal decision-making behavior in different states, help reduce cognitive bias through rational analysis of current states, achieve maximum life benefits, and enrich empirical research in the fields of future self-continuity and intertemporal decision-making.

## 1 Introduction

An individual’s life involves decisions in many areas, such as weighing consumption desires and financial planning, balancing appetite and health, and considering financial interests versus ecological balance. These choices not only affect individuals but also have a broad impact on many disciplines, including economics, psychology, policy, and neuroscience (Ainslie, 1975; Laibson, 1997; Peters and Büchel, 2011; Reeck et al., 2017). People face the choice between small, immediate gains and larger, future returns. This phenomenon is known as intertemporal choice, where individuals must weigh the outcomes of gains or losses at different points in time (especially now and in the future) to make decisions (Frederick et al., 2002; Chen and He, 2012). Numerous studies have shown that the key factors influencing intertemporal decision-making include the decision object, the decision maker, and the external environment (Frederick et al., 2002; Read et al., 2005; Weber et al., 2007; Anokhin et al., 2011). When making intertemporal decisions, people tend to assign less importance to future losses or gains. This phenomenon, known as time discounting, refers to the decrease in the subjective value of delayed outcomes as the delay time increases (Frederick et al., 2002). The magnitude of the time discount rate reflects an individual’s perception of the value of present versus future outcomes and is considered a significant psychological trait (Green and Myerson, 2004). A larger discount rate indicates a greater inclination to choose immediate and smaller benefits, often viewed as more “short-sighted.” In contrast, a smaller discount rate suggests a greater likelihood of choosing delayed but larger returns, which is generally perceived as more rational (Loewenstein and Prelec, 1992; Kalenscher and Pennartz, 2008; Peters and Büchel, 2011; Chen and He, 2012).

Advances in technology and medicine have dramatically increased life expectancy, prompting individuals to consider the future more extensively than in the past. Future self-continuity is defined as the extent to which individuals perceive continuity, identity, and consistency between their present and future selves, significantly influencing intertemporal decision-making (Hershfield H. E. et al., 2009; Liu et al., 2018; Sun et al., 2023). An individual’s perception of their future self and the aspirations they hold for their future identity significantly influence their time discount rate in intertemporal decision-making (Hershfield, 2011; Urminsky, 2017). The self-continuity model posits that individuals are viewed as continuous entities, and the degree of connection between their present and future selves dictates their willingness to sacrifice for the future in intertemporal decision-making, facilitating more favorable and rational decisions (Rutchick et al., 2018; Hershfield, 2019). Despite the clear importance of future self-continuity in intertemporal decision-making, its underlying mechanisms remain inadequately explored. Thus, building on previous studies, this research investigates the influence of future self-continuity on intertemporal decision-making, along with the moderating role of self-concept clarity and the mediating role of future outcome consideration. We propose a mediated regulatory model to elucidate the relationship between future self-continuity and intertemporal decision-making.

Firstly, the influence of future self-continuity on intertemporal decision-making is primarily evident in individuals’ preferences for short-term and long-term options, leading to changes in time discounting (Hershfield H. et al., 2009; Hershfield H. E. et al., 2009). Specifically, individuals with high levels of future self-continuity have a stronger sense of identity with their future selves, are more inclined to make long-term plans, reduce the current time discount rate, and are more willing to choose delayed but greater rewards. Conversely, individuals with a weak sense of future self-continuity may be more inclined to pursue immediate gratification, increase the current time discount rate, and prefer immediate, smaller rewards (Hershfield H. E. et al., 2009). Hershfield (2011) observed that enhancing an individual’s sense of future self-continuity can reduce their time discount rate in intertemporal decision-making. By guiding individuals to focus more on the imagination and meaning of their future selves, their time discount rate in intertemporal decision-making can be effectively reduced, making them more willing to wait for greater returns. Research has shown that time distance and psychological distance increase when considering the distant future (Hershfield H. et al., 2009; Liu et al., 2018). In intertemporal decision-making, a longer time distance increases the psychological distance between the future self and the present self. Psychological distance is a crucial factor contributing to the differences in decision-making between the present self and the future self (Hershfield H. et al., 2009; Hershfield, 2011). Individuals often struggle to maintain a consistent sense of self across different time frames. Uncertainty regarding future options intensifies the psychological distance between one’s present and future selves. Those with strong future self-continuity minimize this temporal gap and reduce the psychological separation between different temporal selves, influencing decision-making processes to prioritize long-term interests and goals. Conversely, individuals with weak future self-continuity perceive their future selves as strangers, tend to prioritize resources for their present selves over their future selves, and are less inclined to sacrifice present satisfaction for future benefits (Liu et al., 2018). Research has found that the degree of psychological connection between an individual and their future self can predict their intertemporal decision-making choices. When individuals perceive their future self as more similar and connected to their present self, they are more inclined to make patient choices, exhibit lower time discount rates, and are more willing to make intertemporal decisions for the benefit of their future self (Bartels and Rips, 2010). To sum up, individuals with high future self-continuity are more inclined to engage in behaviors conducive to long-term interests, while individuals with low future self-continuity are more likely to succumb to the temptation of immediate interests. The influence of future self-continuity on intertemporal decision-making is closely related to various aspects, such as social behavior, charitable donations, and physical health. Individuals with low future self-continuity, who perceive their present self as dissimilar to their future self, are more likely to engage in unethical behavior, such as lying for immediate gain, without much consideration for the effect of such behavior on their future self (Hershfield et al., 2012; Van Gelder et al., 2013). Individuals with high future self-continuity believe that the present self is highly connected to their future self and are more inclined to save or donate to charitable organizations (Zhang and Aggarwal, 2015; Macrae et al., 2017). Rutchick et al. (2018) found that enhancing individuals’ future self-continuity increased participants’ physical exercise behaviors, indicating that future self-continuity can promote behaviors beneficial to individuals’ long-term health. These studies have found that individuals with high future self-continuity exhibit a lower time discount rate than individuals with low future self-continuity (Hershfield H. et al., 2009; Hershfield H. E. et al., 2009). Thus, we proposed Hypothesis 1: Future self-continuity significantly predicts a lower time discount rate in intertemporal decision-making.

Secondly, what are the boundary conditions for future self-continuity to affect intertemporal decision making? Research indicates that low self-concept clarity correlates with low future self-continuity, disrupting the continuity between selves (Slotter and Walsh, 2017; Jiang et al., 2020, 2023). Self-concept clarity refers to the extent to which an individual has a clear, confident, well-defined definition of themselves, as well as internal consistency and periodic stability (Campbell, 1990). The development of self-concept clarity precedes the development of future self-continuity, and the formation of a clear self-concept early in life can promote the development of self-continuity later (Jiang et al., 2020). Studies have found that individuals with low self-concept clarity inhibit self-control by reducing overall self-continuity, which indirectly emphasizes the importance of improving self-concept clarity in coping with self-control failures (Jiang et al., 2023). Markus and Nurius (1986) proposed the “possible self” theory, which holds that the future self is often seen as a possible self, including the self-desired to become and the self-desired to avoid (Markus and Nurius, 1986). A person’s current self-perception and future expectations can significantly influence their intertemporal decision-making (Markus and Kunda, 1986). In intertemporal decision making, varying levels of self-concept clarity can impact the formation of delay discounting behavior, consequently influencing individual choices (Thaler and Shefrin, 1981; Hershfield, 2011). The multiple self-concept model proposes that individuals may have multiple competing selves representing different interests and preferences when making decisions. These competing selves may lead individuals to make inconsistent or irrational decisions in various situations, such as “short-sighted” doers and “long-sighted” planners (Thaler and Shefrin, 1981). When an individual has a clear and specific image of themselves in the future, it promotes them to make more rational and favorable decisions for the future (Hershfield, 2011). In summary, individuals with different levels of future self-continuity tend to choose delayed and more advantageous intertemporal decisions, a tendency that may be based on higher levels of self-clarity. For individuals with high levels of self-clarity, those with higher future self-continuity can more clearly recognize their current and future needs compared to those with low future self-continuity, making it easier for them to choose delayed and more profitable intertemporal decisions. However, individuals with a low level of self-clarity may struggle to understand their own needs clearly and be less aware of the distinction between the present and the future, leading to an unclear relationship between their future self-continuity and intertemporal decision-making. Thus, we proposed Hypothesis 2: In the influence of future self-continuity on intertemporal decision-making, clarity of future self-concept plays a moderating role.

Thirdly, how does future outcome consideration play a mediating role in the moderating effect of self-concept clarity on future self-continuity and intertemporal decision-making? Individual differences exist in the extent to which individuals emphasize future outcome consideration in intertemporal decision-making. Studies have shown that individuals’ future outcome consideration can influence their information processing of current behaviors, as well as their attitude and intention formation, thus affecting intertemporal decision-making (Strathman et al., 1994; Kim and Nan, 2016; Pozolotina and Olsen, 2019). Future outcome consideration refers to the degree to which individuals contemplate the possible long-term consequences of their actions and how much they are influenced by those potential outcomes (Strathman et al., 1994; Joireman and King, 2016). Rappange et al. (2010) demonstrated a significant correlation between future outcome consideration and time discount rates. When individuals with a low level of future outcome consideration make intertemporal decisions, they perceive future information as unconvincing and current information as more important. They also believe that the certainty and concreteness of immediate goals are more impactful than the probability and abstractness of future goals. Studies have found a significant correlation between lower consideration of future outcomes and behaviors such as binge drinking and a greater preference for immediate gratification (McKay et al., 2013). Studies have shown that when distributing income, individuals with higher future outcome consideration pay more attention to their future selves and are more inclined to invest and save for retirement (Howlett et al., 2008). Future outcome consideration reflects an individual’s concern for the future consequences of decision-making behavior. Individuals with low levels of future outcome consideration focus more on immediate results, while those with high levels focus more on the future impact of their decisions. Research indicates that individuals with high future self-continuity are better able to imagine the future impact of their current behavior, value future outcomes more, and are more willing to consider the long-term benefits of delayed choices. Conversely, individuals with low future self-continuity believe the connection between their present self and future self is weak. Their consideration of future results is more vague, causing them to underestimate the impact of current choices on the future and focus more on immediate satisfaction (Strathman et al., 1994). In conclusion, future outcome consideration is a critical factor influencing individuals’ intertemporal decision-making. Individuals with a lower levels of future outcome consideration focus more on immediate results, leading to a higher discount rate. Conversely, individuals with high levels of future outcome consideration are more concerned with future returns, resulting in a lower discount rate (Kim and Nan, 2016). Thus, we proposed Hypothesis 3: Self-concept clarity and future outcome consideration have mediating moderating effects on the relationship between future self-continuity and intertemporal decision-making.

This study aimed to explore the influence of future self-continuity on intertemporal decision-making and to examine the moderating effect of self-concept clarity and the mediating effect of future outcome consideration. To achieve this, two studies were designed. Study 1 utilized a questionnaire to investigate the relationship between future self-continuity and intertemporal decision-making, as well as to examine the moderating role of self-concept clarity. Study 2 employed an experimental method, dividing participants into high and low future self-continuity groups, to further explore the mediating effect of future outcome consideration and the moderating role of self-concept clarity in the influence of future self-continuity on intertemporal decision-making. Through these two studies, we aimed to reveal how future self-continuity, by influencing individuals’ consideration of future outcomes and the clarity of their self-concept, alters intertemporal decision-making behavior, thereby providing new insights into understanding and improving decision-making processes.

## 2 Study 1

### 2.1 Method

#### 2.1.1 Participants

In this study, a total of 502 questionnaires were distributed through the sampling service provided by the website “Questionnaire Star”^1^. During the study, three attention test questions were included (e.g., please select “fully agree” for the attention test question, Ren et al., 2022). A total of 132 subjects failed one or more attention test questions. After excluding invalid responses, a total of 370 valid questionnaires were used in this study, comprising 264 from female participants and 106 from male participants, with an effective response rate of 73.71%. Participants ranged in age from 18 to 35 years (*M* = 19.54, *SD* = 3.02). This study was conducted in accordance with the Declaration of Helsinki and approved by the Ethics Committee. Participants provided informed consent. Participants completed the questionnaires, received compensation, and were assured that the data collected would be used only for this study.

#### 2.1.2 Materials and experimental task

##### 2.1.2.1 Future self-continuity questionnaire

The Future Self-Continuity Questionnaire (FSCQ) developed by Sokol and Serper (2020) is adopted to measure an individual’s perceived degree of continuity and consistency between the present self and the future self (Hershfield, 2011). The questionnaire consists of 10 items, divided into three subscales: similarity, vividness and positivity. The similarity subscale comprises four items (e.g., “How similar will you be now to who you will be 10 years from now,” “How similar will your thoughts be now to what you will be 10 years from now”). The vividness consists of three items (e.g., “How vividly can you imagine yourself 10 years from now?” and “How vividly can you imagine your family relationship 10 years from now?”). The positivity contains three items (e.g., “How much do you like yourself 10 years from now,” “How much do you like the way you do things 10 years from now”). Participants’ responses were scored using a 7-point Likert scale (1 = *completely inconsistent*, 7 = *completely consistent*). A higher total score indicates a higher level of future self-continuity for an individual. This scale has good reliability and validity among Chinese youth and is used to assess future self-continuity in youth (Zhang et al., 2022). The Cronbach’s alpha of this questionnaire in this study is 0.900, and the Cronbach’s alpha of the similarity, vividness, and positivity sub-questionnaires were 0.854, 0.722, and 0.893, respectively.

##### 2.1.2.2 Self-concept clarity scale

The Self-Concept Clarity Scale (SCCS) was used to measure the clarity and consistency of individual self-concept (Campbell et al., 1996). The scale comprises 12 items, with examples such as “Sometimes I have one view of myself, sometimes I have a different view of myself” and “Generally speaking, I have a clear idea of who I am and what kind of person I am.” It is important to note that, except for item 6 (“I rarely experience conflict between different aspects of my personality”) and item 11 (“Generally speaking, I have a clear idea of who I am and what I am like”), all questions are reverse-scored. Responses were rated on a 7-point scale (1 = *completely inconsistent*, 7 = *completely consistent*), with higher scores indicating a higher level of self-concept clarity. Previous studies have demonstrated that the Chinese version of the questionnaire has good reliability and validity (Liu et al., 2017). The Cronbach’s alpha of the self-concept clarity scale in this study was 0.841.

##### 2.1.2.3 The monetary-choice questionnaire

In this study, we adopted the currency selection paradigm proposed by Kirby et al. (1999). Before the study, participants were explicitly told that they would be involved in a real decision-making task. They were told that only those who completed the entire experiment and were consistently involved were eligible for the reward. We used a questionnaire in which participants were asked to make 27 fixed choices between smaller, immediate rewards (SIRs) and larger, delayed rewards (LDRs). Each item has two options: Option A and Option B. Option A refers to smaller, immediate rewards. Option B refers to the larger, varying number of days of delay earned rewards. Participants were asked to choose each item based on their true preferences. For example, participants were asked, “Would you prefer to get $238 today or $245 in 186 days?” The participant marks the options he or she would like to accept on the questionnaire. Values for all 27 trials are shown in Supplementary material. To encourage accurate responses, participants were offered two types of compensation: a basic fee for participating in the experiment and an additional fee based on their performance on the currency choice questionnaire. At the end of the trial, participants were randomly selected one of 27 trials (at a 1% rate) as the final additional participant fee. If the selected option is immediate, the participant receives the corresponding amount immediately. If the selected option is delayed (different days), and if the participant chooses the larger delayed reward, the corresponding amount will be received 1 week later. The process is carried out using pen and paper measurements.

##### 2.1.2.4 Objective socio-economic status

Objective socio-economic status was controlled for in this study due to its involvement in economic decision-making processes. It was measured using *per capita* monthly household income (Griskevicius et al., 2013; Mittal and Griskevicius, 2014) and scored on a 9-point scale (1 = *￥1,500 and below*, 2 = *￥1,501 ~ 2,500*, 3 = *￥2,501 ~ 3,500*, 4 = *￥3,501 ~ 5,000*, 5 = *￥5,001 ~ 7,500*, 6 = *￥7,501 ~ 10,000*, 7 = *￥10,001 ~ 15,000*, 8 = *￥15,001 ~ 20,000*, 9 = *￥20,001 and above*), with higher scores indicating higher objective socioeconomic status.

#### 2.1.3 Data recording and analysis

All participants used the “Questionnaire Star” website to complete the survey and complete it independently within the specified time (about 10 min) according to their personal situation. Data for this study were obtained after excluding invalid questionnaires. Descriptive statistics and correlation analysis were conducted using SPSS 25.0, and a structural equation model was established using Mplus 8.3 for further analysis. In these analyses, future self-continuity was added as a latent independent variable, intertemporal decision-making as a latent dependent variable, self-concept clarity as a latent moderating variable, and age, gender, and objective socioeconomic status as control variables in the model.

### 2.2 Results

#### 2.2.1 Common method variance test

In the process of questionnaire survey, anonymous measurement and partial item reverse were adopted to control the common method deviation (Zhou and Long, 2004). Therefore, in order to ensure the validity of the measurement results, we conducted Harman single factor test to check the common method bias, and conducted exploratory factor analysis on all measurement data. The results showed that a total of 10 unrotated common factors with eigenvalues greater than 1 were obtained, and the variance explanatory rate of the maximum factor was 18.466%. Therefore, there was no significant common method bias in this study (Podsakoff et al., 2003).

#### 2.2.2 Describe statistics and related analysis

Table 1 presented the mean, standard deviation, and Pearson correlation matrix for each variable. The correlation analysis results indicated a significant negative correlation (*r* = −0.163, *p* = 0.002) between future self-continuity and the time discount rate of intertemporal decision-making, and a significant positive correlation (*r* = 0.102, *p* = 0.050) with self-concept clarity. However, the time discount rate for clear self-concept and intertemporal decision-making was not significant (*r* = 0.053，*p* = 0.307).

**Table 1:** Descriptive statistics and correlations for tested variables in Study 1.

Variables	*M*	*SD*	1	2	3	4	5	6
1. Gender	0.290	0.453	—					
2. Age	19.540	3.019	0.139**	—				
3. OSES	5.640	1.912	0.094	0.264***	—			
4. FSC	4.542	0.972	0.025	0.068	0.142**	—		
5. SCC	3.692	0.946	0.023	0.230***	0.135**	0.102*	—	
6. TDR	0.027	0.052	0.008	−0.042	−0.041	−0.163**	0.053	—

M, mean; SD, standard deviation, ***p < 0.001, **p < 0.01, *p < 0.05. OSES, objective socio-economic status; FSC, future self-continuity; SCC, self-concept clarity; TDR, time discount rate.

#### 2.2.3 Main effect model analysis

To verify the main effect of future self-continuity on intertemporal decision-making, establish a structural equation model using Mplus 8.3 (Wu and Wen, 2011). This model includes one latent independent variable and one latent dependent variable. The measurement model fit the criteria well (*χ^2^* = 0.768, *df* = 2, RMSEA = 0.001, CFI = 1.000, TLI = 1.000, SRMR = 0.008), with factor loadings ranging from 0.757 to 0.811.

After all measures were standardized and gender, age and objective socioeconomic status were included as control variables, the fit of the main effect model was satisfactory (*χ^2^* = 16.869, *df* = 11, RMSEA = 0.038, CFI = 0.985, TLI = 0.976, SRMR = 0.051). It was found that future self-continuity significantly negatively predicted the time discount rate in intertemporal decision-making (*b* = −0.217, *p* = 0.002, 95%CI = [−0.357, −0.078]). The results indicated that future self-continuity significantly and negatively predicted the time discount rate of intertemporal decision-making (*b* = −0.154, *p* = 0.013, 95%CI = [−0.277, −0.032]).

#### 2.2.4 Moderation model analysis

**Figure 1:**
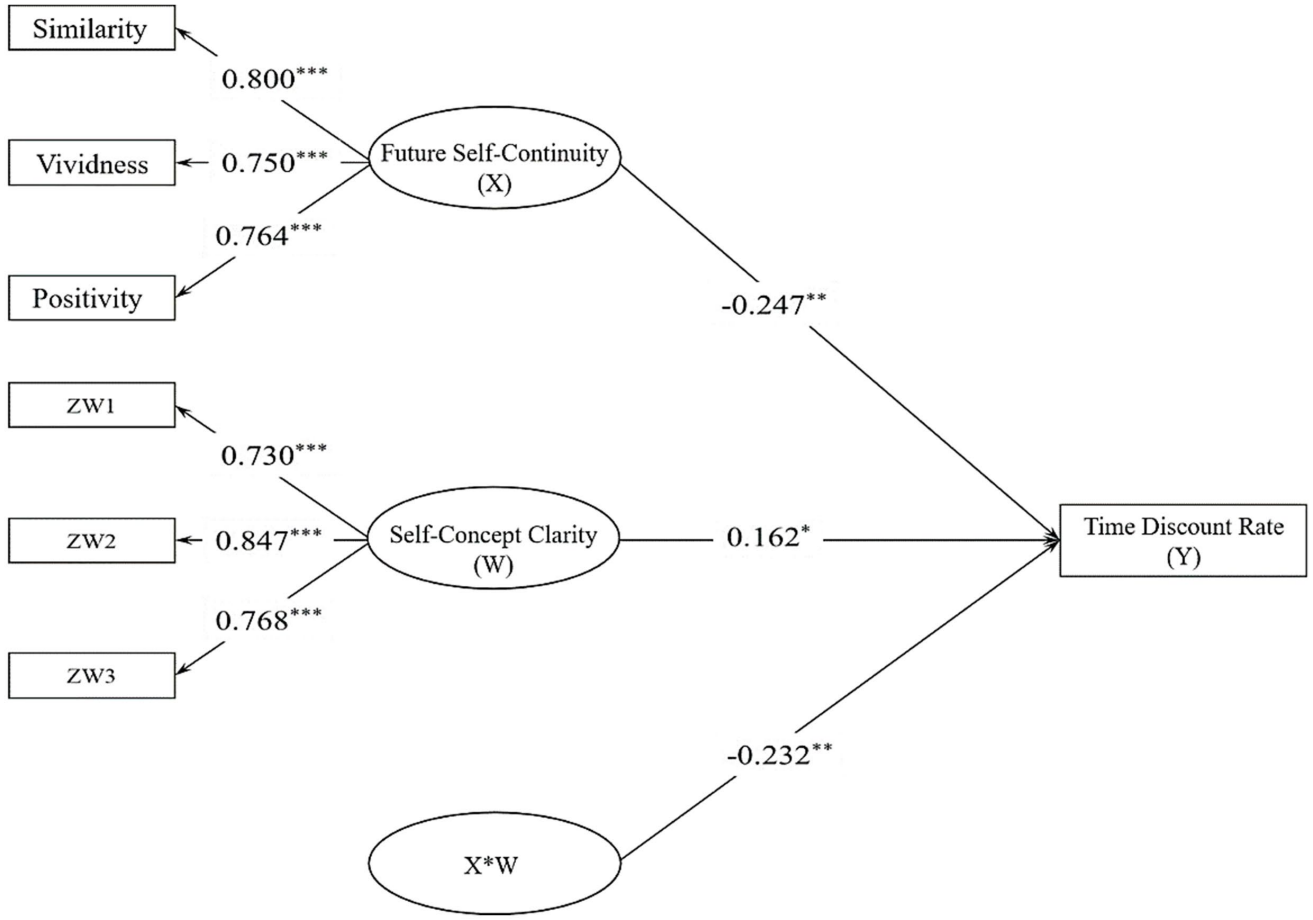
Moderation model of Study 1 (*p < 0.05, **p < 0.01, ***p < 0.001). ZW = a measure of self-concept clarity.

A structural equation model was established to verify the moderating effect of self-concept clarity on the relationship between future self-continuity and intertemporal decision-making. The model includes one latent independent variable, one latent moderating variable, and one latent dependent variable, as shown in Figure 1. The measurement model fit the criteria well (*χ^2^* = 47.582, *df* = 12, RMSEA = 0.090, CFI = 0.958, TLI = 0.926, SRMR = 0.050), with factor loadings ranging from 0.733 to 0.847.

Due to the latent nature of both the independent and moderating variables in this study, maximum likelihood estimation requires numerical integration, leading to a substantial computational burden (Asparouhov and Muthén, 2021) and posing challenges for the convergence of the moderating model. Consequently, this study employs a combination of the latent structural equation method and Bayesian method to construct a regulatory model and derive Bayesian estimates of the regulatory effect (Asparouhov and Muthén, 2021; Fang and Wen, 2022; Ozkok et al., 2022). As demonstrated in the simulation study by Asparouhov and Muthén (2021), Bayesian estimation, in the analysis of regulatory effects using the latent regulatory structural equation method, is faster and more accurate than the maximum likelihood estimation employed by Preacher et al. (2016), with smaller absolute deviation, better inter region coverage, and a higher convergence rate. The Bayesian method relies on the convergence of the Markov Chain, where a PSR (Potential Scale Reduction) < 1.1 indicates convergence. In the moderating model of this study, the PSR results were reported as PSR = 1.007 at the 2000th iteration, indicating Markov chain convergence (Fang and Wen, 2022).

This study standardized all measurement indicators and added gender, age and objective socio-economic status as control variables into the model. The results of the moderation model found that future self-continuity significantly negatively predicted the time discount rate of intertemporal decision-making (*b* = − 0.247, *p_one-tailed_* = 0.002, 95%CI = [−0.399, −0.104]). The clarity of self-concept significantly positively predicted the time discount rate of intertemporal decision-making (*b* = 0.162, *p_one-tailed_* = 0.022, 95%CI = [0.002, 0.323]). Furthermore, the clarity of self-concept significantly negatively moderated the relationship between future self-continuity and the time discount rate of intertemporal decision-making (*b* = − 0.232, *p_one-tailed_* = 0.007, 95%CI = [−0.427, −0.048]).

**Figure 2:**
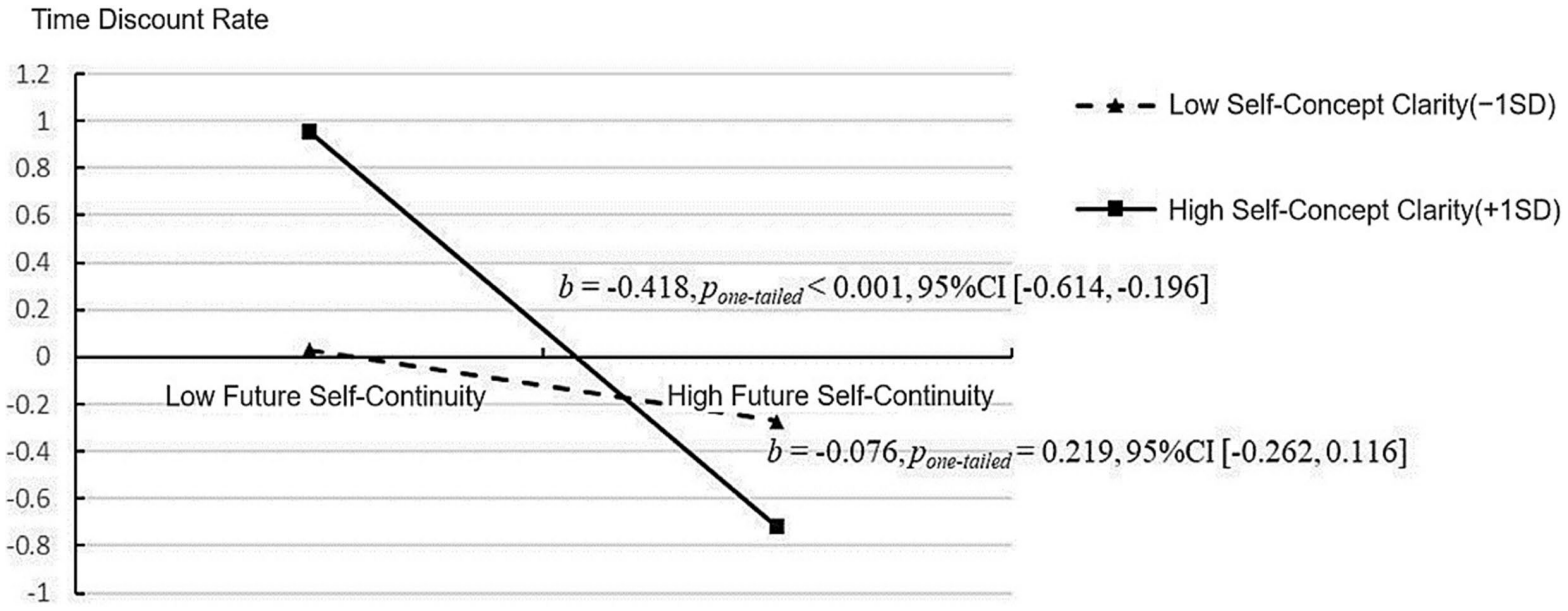
Simple effect analysis results of Study 1.

This study conducted a simple slope analysis to better understand the regulatory role of self-concept clarity, as depicted in Figure 2. The results showed that in individuals with lower levels of self-concept clarity (−1SD), the prediction of future self-continuity on the time discount rate of intertemporal decision-making was not statistically significant (*b* = −0.076, *p_one-tailed_* = 0.219, 95%CI = [−0.262, 0.116]). Conversely, in individuals with a high level of self-concept clarity (+1SD), future self-continuity significantly negatively predicts the time discount rate of intertemporal decision-making (*b* = −0.418, *p_one-tailed_* < 0.001, 95%CI = [−0.614, −0.196]).

### 2.3 Discussion of study 1

The research findings indicated that future self-continuity could significantly negatively predict the time discount rate of intertemporal decision-making, which is consistent with existing research findings (Bartels and Rips, 2010; Macrae et al., 2017). The self-continuity model of intertemporal decision-making suggests that individuals have a subjective perception of the connection between their current and future selves at different time points. When individuals feel closely connected to their future selves (high future self-continuity), they are more likely to consider the future, make decisions that benefit their future selves, and exhibit a preference for delayed rewards (Hershfield H. E. et al., 2009), which verified hypothesis 1.

In addition, the research findings found that individual self-concept clarity could significantly regulate the relationship between future self-continuity and intertemporal decision-making, which is consistent with existing research findings (Thaler and Shefrin, 1981; Hershfield, 2011; Jiang et al., 2023). Only in individuals with high levels of self-concept clarity could future self-continuity significantly negatively predict the time discount rate of intertemporal decision-making; in individuals with lower levels of self-concept clarity, there was no significant relationship between future self-continuity and the time discount rate of intertemporal decision-making, which verified hypothesis 2. Perhaps it is because individuals with high self-concept clarity could have a clearer understanding and comprehension of their self-concept, and could have a clearer understanding of the life outcomes they want (Campbell, 1990). Therefore, they could make intertemporal choices based on their perceived closeness to the present and future. For individuals with low self-concept clarity, confusion in their self-concept cognition could lead to self-interruption, making it difficult to clearly understand their life goals (Costin and Vignoles, 2020), resulting in a lack of significant relationship between future self-connectedness and intertemporal decision-making.

## 3 Study 2

### 3.1 Method

#### 3.1.1 Participants

Using G-Power 3.1 (Faul et al., 2007) to calculate the required sample size with an effect size of *Cohen’s d* = 0.4, and setting *α* = 0.05, at least 200 participants were required to reach 80% (1−*β*). The statistical test showed that 262 college students were actually recruited. During the study, three attention test questions were included (e.g., if this question is an attention test question, please choose “completely disagree,” Ren et al., 2022). A total of 28 participants did not pass the attention test for one or more questions. After excluding invalid participants, 234 participants (89 males, *M* = 18.71, *SD* = 1.40) were included in the final analysis. The number of participants in the high and low future self-continuity groups was 117 each. All participants were right-handed, with normal binocular vision or corrected vision, and had not participated in similar experiments. This study was conducted in accordance with the Helsinki Declaration and was approved by the Ethics Committee. Participants provided informed consent. They completed the questionnaire, received compensation, and were assured that the collected data would only be used for this study.

#### 3.1.2 Experimental design

Adopting a 2 (future self-continuity: high vs. low) between-subjects experimental design, the independent variable is future self-continuity, the dependent variable is the time discount rate in intertemporal decision-making, the moderating variable is self-concept clarity, the mediating variable is future outcome consideration, and the control variables are gender, age, and objective socio-economic status.

#### 3.1.3 Materials and experimental task

##### 3.1.3.1 Future self-continuity manipulation

Future self-continuity was manipulated using reading and writing tasks (Bartels and Urminsky, 2011; Zhang and Aggarwal, 2015; Liu and Zhang, 2022). Firstly, participants read a piece of material designed to manipulate the level of self-continuity and assessed the relationship between their current self and their self in the next 10 years.

*Scenarios that enhance self-continuity: People’s moral qualities, personality, beliefs, values, etc., will not undergo substantial changes. Although life goes through different stages, each stage is continuous and interconnected. Everyone’s life is a continuous unity.*

*Scenarios that reduce self-continuity: People’s moral qualities, personality, beliefs, values, etc., will undergo substantial changes over time. Life goes through different stages, and each stage is fragmented and unrelated. Everyone’s life is composed of multiple different stages*.

Secondly, after reading the material, participants need to continue with the writing task, describing the similarities or differences between their current self and their future self (Zhang and Aggarwal, 2015). To control the impact of future time distance, the future is set as “10 years later,” and the writing word count is controlled to around 50 words. Participants in the high future self-continuity group need to list, as specifically as possible, the similarities between themselves 10 years later and their current self, while participants in the low future self-continuity group need to list, as specifically as possible, the differences between themselves 10 years later and their current self.

After the manipulation was completed, a manipulation check of future self-continuity was conducted using the same questionnaire as Study 1. During the measurement process, each question was accompanied by a stateful description (“Please rate the following description based on your true thoughts at this moment,” Mittal and Griskevicius, 2014). The Cronbach’s alpha of this questionnaire in this experiment is 0.906, and the Cronbach’s alpha of the similarity, vividness, and positivity sub-questionnaires were 0.851, 0.771, and 0.855, respectively.

##### 3.1.3.2 Future outcome consideration scale

The Future Outcome Consideration Scale was used to measure participants’ state and future outcome considerations, utilizing the future dimensions from the scale (Strathman et al., 1994; Feng et al., 2020). The future dimension of this scale includes 5 items, such as “I will consider what things will be like in the future and try to influence those things through my daily behavior” and “I think it is important to make the worst plan for the future, even if these bad things may not happen for many years in the future.” During the measurement process, each question was accompanied by a stateful description (“Please rate the following description based on your true thoughts at this moment,” Mittal and Griskevicius, 2014). Using a 7-point rating (1 = *completely disagree*, 7 = *completely agree*), the higher the score, the more likely the individual is to consider the future. Previous studies have shown that the Chinese version of the questionnaire has good reliability and validity (Feng et al., 2020). In this study, the Cronbach’s alpha of future outcome consideration was 0.719.

##### 3.1.3.3 Self-concept clarity scale

The Self-Concept Clarity Scale was the same as that used in Study 1 to measure the participants’ self-concept clarity (Campbell et al., 1996). The Cronbach’s alpha for the self-concept clarity scale in this experiment was 0.834.

##### 3.1.3.4 The monetary-choice questionnaire

**Figure 3:**
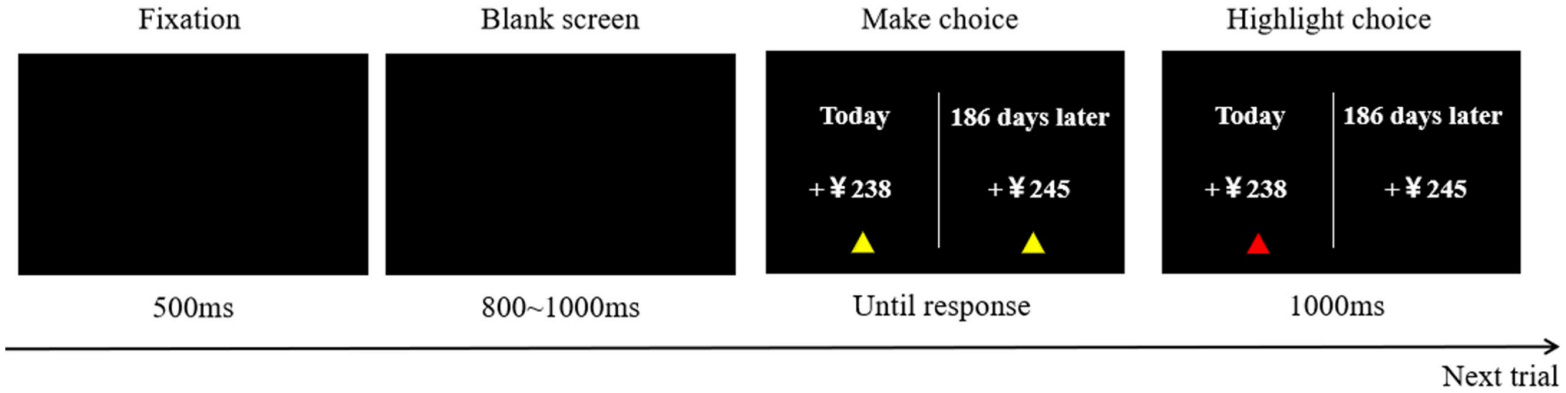
Flow chart of monetary-choice paradigm.

The monetary-choice questionnaire followed the same experimental process as in Study 1, but Study 2 used a computer-generated format for answering, as shown in Figure 3. In each trial, a “+” appeared in the center of the computer screen for 500 ms to remind the participants to start the experiment. Then, two options appeared on the screen. The left option represented small, immediate rewards (SIRs), with the amount obtainable immediately. The right option represented large, delayed rewards (LDRs), with the amount obtainable after a certain period of time (a few days), requiring the participants to make choices based on their true thoughts. Participants selected the left option by pressing the “F” key and the right option by pressing the “J” key. After pressing the button, a small triangle appeared below the selected option for 1,000 ms to confirm the selection, and then the experiment proceeded to the next trial. The entire task comprised 27 trials.

##### 3.1.3.5 Objective socio-economic status

Objective socio-economic status was controlled for in this study due to its involvement in economic decision-making processes. It was measured using *per capita* monthly household income (Griskevicius et al., 2013; Mittal and Griskevicius, 2014) and scored on a 9-point scale (1 = *￥1,500 and below*, 2 = *￥1,501 ~ 2,500*, 3 = *￥2,501 ~ 3,500*, 4 = *￥3,501 ~ 5,000*, 5 = *￥5,001 ~ 7,500*, 6 = *￥7,501 ~ 10,000*, 7 = *￥10,001 ~ 15,000*, 8 = *￥15,001 ~ 20,000*, 9 = *￥20,001 and above*), with higher scores indicating higher objective socioeconomic status.

#### 3.1.4 Materials and experimental task

All participants completed the experiment in a separate small room, which consisted of three main parts. Firstly, all participants completed the Self Concept Clarity Scale through an online questionnaire platform. Secondly, the participants completed reading and writing tasks and conducted operational tests on the grouping of state-based self-continuity and measurement of state-based future outcomes. We divided the participants into high self-continuity and low self-continuity groups. Finally, all participants completed the monetary-choice paradigm, and behavioral data were collected using E-prime 2.0 software (Psychological Software Tool, Pittsburgh, Pennsylvania, United States). To ensure that all participants understood the experimental procedure, practice experiment was conducted before the formal experiment began. Participants were informed that the experiment involved two real-life scenarios, each with 27 sets of options. They were instructed to carefully pay attention to the differences between options and make a choice after thorough consideration.

#### 3.1.5 Data recording and analysis

Behavioral data were analyzed using SPSS 25.0 (IBM, Armonk, NY, United States). Firstly, independent sample *t*-test [2 (future self-continuity: high vs. low)] was used to compare the future self-continuity scores under different conditions. Secondly, descriptive statistics and correlation analyses were conducted on the data. A structural equation model was established using Mplus 8.3 for model analysis. In these analyses, future self-continuity was considered as the explicit independent variable, intertemporal decision-making as the latent dependent variable, self-concept clarity as the latent moderating variable, and future outcomes as the latent mediating variable. Age, gender, and objective socio-economic status were included in the model as control variables.

### 3.2 Results

#### 3.2.1 Manipulation test

In the operational test of future self-continuity, individuals in the high future self-continuity group reported significantly higher future self-continuity scores (*M* ± *SD* = 5.113 ± 0.690) than those in the low future self-continuity group (*M* ± *SD* = 3.604 ± 0.629), *t*(232) = −17.479, *p* < 0.001, Cohen’s *d* = 2.286, 95% CI = [−1.678, −1.339], indicating that the manipulation of future self-continuity in this experiment was effective.

#### 3.2.2 Describe statistics and related analysis

Table 2 presented the mean, standard deviation, and Pearson correlation matrix for each variable. The correlation analysis revealed a significant negative correlation between future self-continuity and the time discount rate of intertemporal decision-making (*r* = −0.150, *p* = 0.022), as well as a significant positive correlation between future self-continuity and future outcomes (*r* = 0.312, *p* < 0.001). However, there was no significant correlation between future self-continuity and self-concept clarity (*r* = −0.048, *p* = 0.463). Additionally, the consideration of future outcomes showed significant negative correlations with the time discount rate (*r* = −0.270, *p* < 0.001) and self-concept clarity (*r* = −0.375, *p* < 0.001) in intertemporal decision-making. The correlation between the time discount rate and clear self-concept in intertemporal decision-making was not significant (*r* = 0.086, *p* = 0.189).

**Table 2:** Describes the statistical and related analysis results in Study 2.

Variables	*M*	*SD*	1	2	3	4	5	6	7
1. Gender	0.380	0.487	—						
2. Age	18.710	1.397	0.090	—					
3. OSES	4.590	1.938	0.163*	−0.161*	—				
4. FSC	0.500	0.501	−0.044	0.034	0.055	—			
5. FOC	4.377	0.918	−0.067	0.034	0.050	0.312***	—		
6. SCC	3.806	0.917	0.151*	0.020	−0.013	−0.048	−0.375**	—	
7. TDR	0.036	0.068	0.193**	0.194**	0.066	−0.150*	−0.270**	0.086	—

M, mean; SD, standard deviation, ***p < 0.001, **p < 0.01, *p < 0.05. OSES, objective socio-economic status; FSC, future self-continuity; FOC, future outcome consideration; SCC, self-concept clarity; TDR, time discount rate.

#### 3.2.3 Main effect model analysis

Conduct path analysis using Mplus 8.3 to confirm the main effect of future self-continuity on intertemporal decision-making. Upon including gender, age, and objective socio-economic status as control variables in the model, the main model reached saturation. The results indicated that future self-continuity significantly and negatively predicted the time discount rate of intertemporal decision-making (*b* = −0.154, *p* = 0.013, 95%CI = [−0.277, −0.032]).

#### 3.2.4 Moderation model analysis

**Figure 4:**
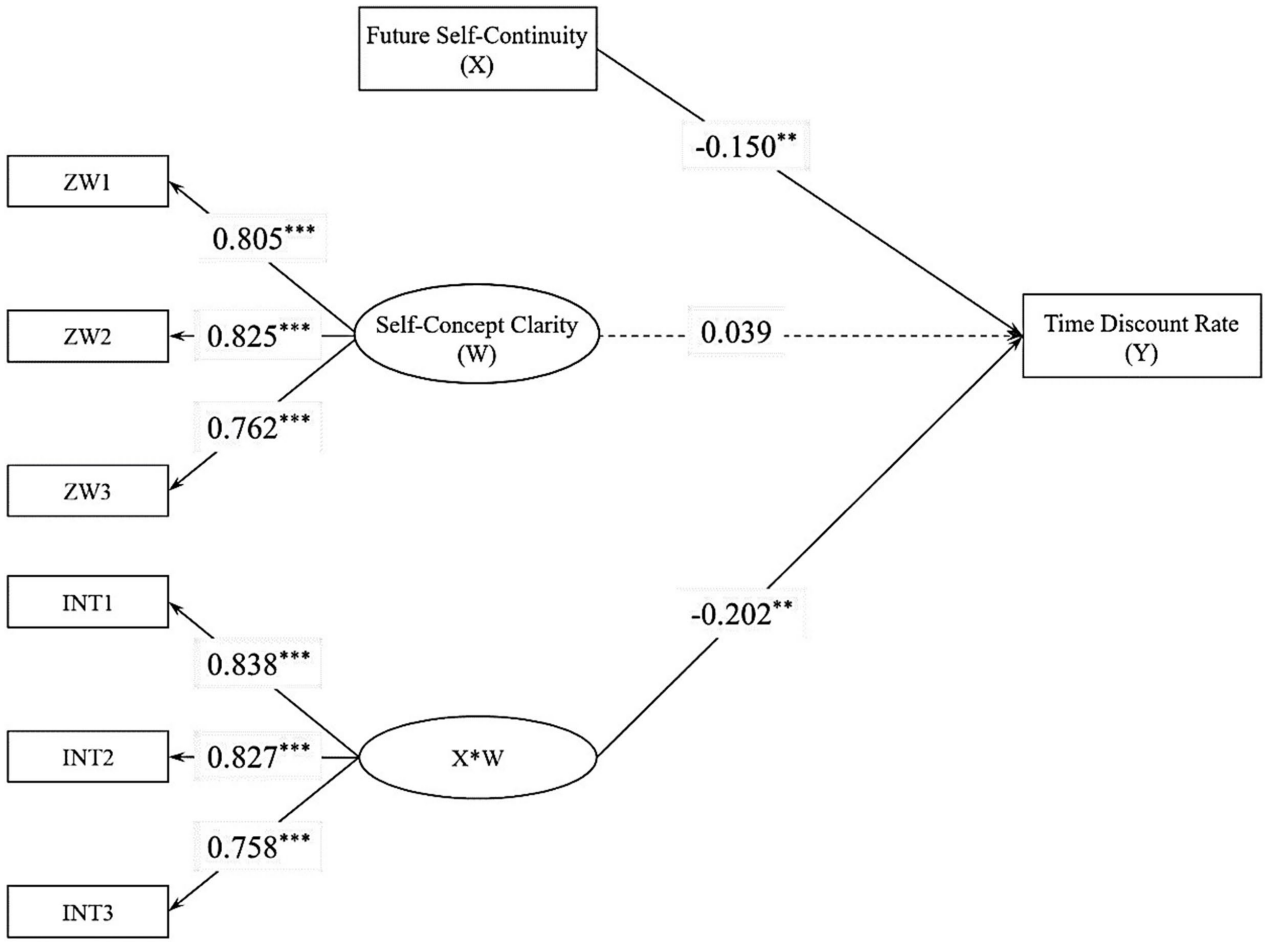
Moderation model of Study 2. **p < 0.01, ***p < 0.001. ZW = a measure of self-concept clarity; INT = measurement metric of the interaction term.

Construct a structural equation model using Mplus 8.3 to examine the moderating effect of self-concept clarity on the relationship between future self-continuity and intertemporal decision-making (1 explicit independent variable, 1 latent moderating variable, and 1 explicit dependent variable, refer to Figure 4 for the model). During model development, the measurement indicators are also incorporated using the project balance method. The measurement model fit has reached saturation, with factor loadings ranging from 0.766 to 0.833. Interaction terms were constructed using the product index method (Wen et al., 2003; Wu et al., 2011; Wen et al., 2022), and a moderation model was established using Bayesian methods to obtain Bayesian estimates of the moderation effect (Asparouhov and Muthén, 2021; Fang and Wen, 2022; Ozkok et al., 2022). In this study’s moderation model, the PSR results indicated a PSR of 1.006 at the 2000th iteration, indicating the convergence of the Markov chain (Fang and Wen, 2022).

After standardizing all measurement indicators and including gender, age, and objective socio-economic status as control variables in the model, the results of the adjustment model indicated that future self-continuity significantly negatively affected the time discount rate of intertemporal decision-making (*b* = −0.150, *p_one-tailed_* = 0.009, 95%CI = [−0.277, −0.028]). The effect of self-concept clarity on the time discount rate of intertemporal decision-making was not significant (*b* = 0.039, *p_one-tailed_* = 0.326, 95%CI = [−0.124, 0.221]). However, self-concept clarity significantly moderated the impact of future self-continuity on the time discount rate of intertemporal decision-making (*b* = −0.202, *p_one-tailed_* = 0.007, 95%CI = [−0.356, −0.038]).

To better understand the moderating role of self-concept clarity, this study conducted a simple slope analysis. The results indicated that among individuals with lower levels of self-concept clarity (−1 SD), the impact of future self-continuity on the time discount rate of intertemporal decision-making was not significant (*b* = −0.016, *p_one-tailed_* = 0.428, 95%CI = [−0.153, 0.202]). In contrast, among individuals with higher levels of self-concept clarity (+1 SD), future self-continuity significantly negatively affected the time discount rate of intertemporal decision-making (*b* = −0.317, *p_one-tailed_* < 0.001, 95%CI = [−0.507, −0.143]).

#### 3.2.5 Mediated moderating model

**Figure 5:**
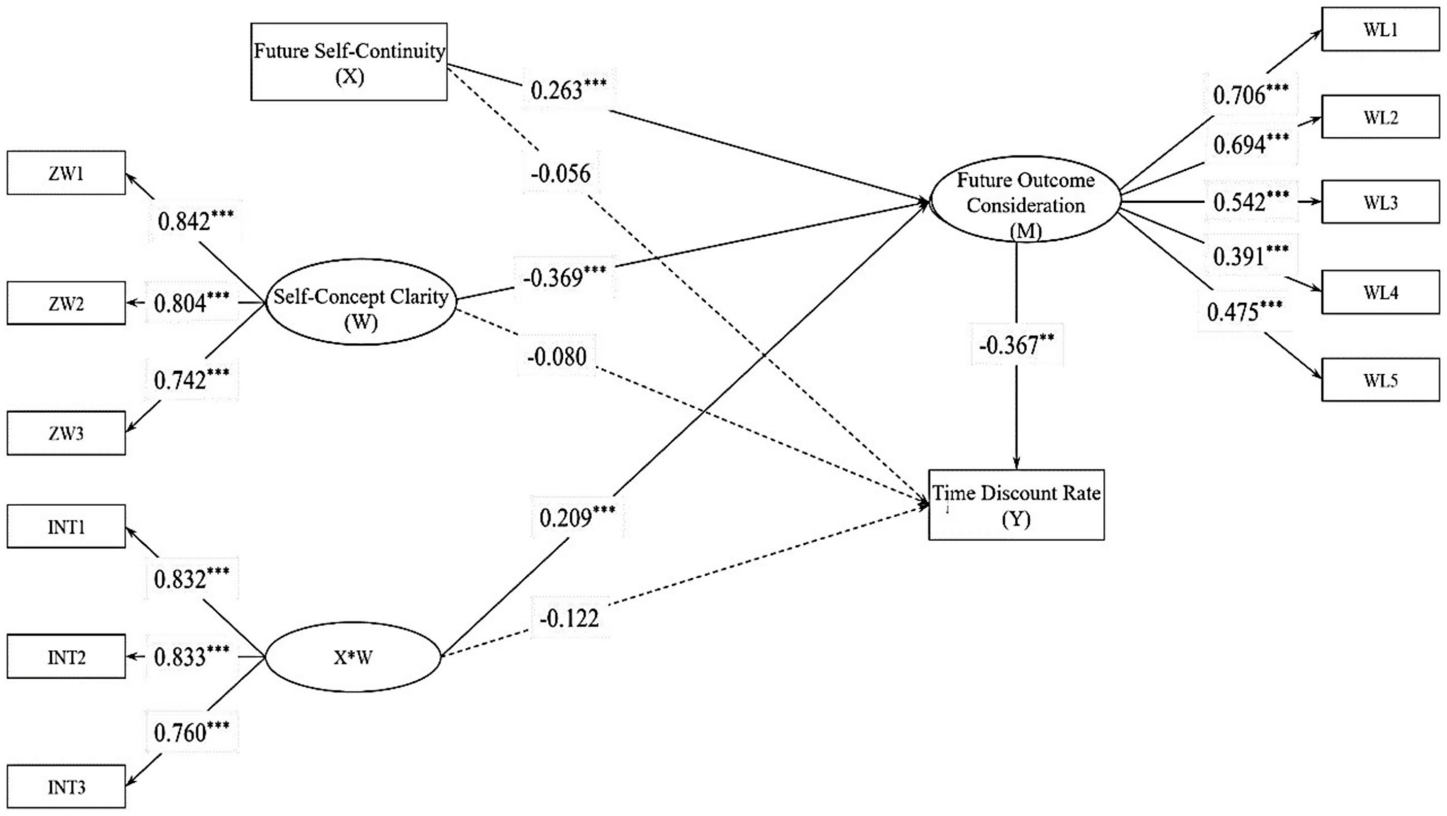
Mediated moderating model in Study 2. *p < 0.05, **p < 0.01, ***p < 0.001. ZW = a measure of self-concept clarity; INT = measurement indicator of interaction term; WL = measurement indicator considered for future outcome.

Establish a structural equation model using Mplus 8.3 to validate the moderated mediation model (1 explicit independent variable, 1 latent moderating variable, 1 latent mediating variable, 1 explicit dependent variable, see Figure 5 for the model). During the modeling process, the measurement indicators were also packaged using the project balance method. The measurement model fit met the standard criteria (*χ^2^* = 71.906, *df* = 19, RMSEA = 0.109, CFI = 0.911, TLI = 0.868, SRMR = 0.058), with factor loadings ranging from 0.434 to 0.876.

Similar to the moderation model, the product index method was used to construct the interaction term, and the Bayesian method was employed to establish a moderated mediation model, obtaining Bayesian estimates of the moderation and mediation effects. In the moderated model of this study, the PSR results indicated a PSR of 1.007 at the 2000th iteration, demonstrating the convergence of the Markov chain (Fang and Wen, 2022).

After standardizing all measurement indicators and incorporating gender, age, and objective socio-economic status as control variables into the model, the moderated model found that the effect of future self-continuity on the time discount rate of intertemporal decision-making was not significant (*b* = −0.056, *p_one-tailed_* = 0.223, 95%CI = [−0.192, 0.087]). Future self-continuity significantly positively influenced future outcome considerations (*b* = 0.263, *p_one-tailed_* < 0.001, 95%CI = [0.167, 0.371]). Future outcome considerations significantly negatively affected the time discount rate of intertemporal decision-making (*b* = −0.367, *p_one-tailed_* = 0.003, 95%CI = [−0.636, −0.060]). Self-concept clarity significantly moderated the impact of future self-continuity on the consideration of future outcomes (*b* = 0.209, *p_one-tailed_* < 0.001, 95%CI = [0.083, 0.337]). However, self-concept clarity did not significantly moderate the impact of future self-continuity on the time discount rate of intertemporal decision-making (*b* = −0.122, *p_one-tailed_* = 0.089, 95%CI = [−0.302, 0.047]).

**Figure 6:**
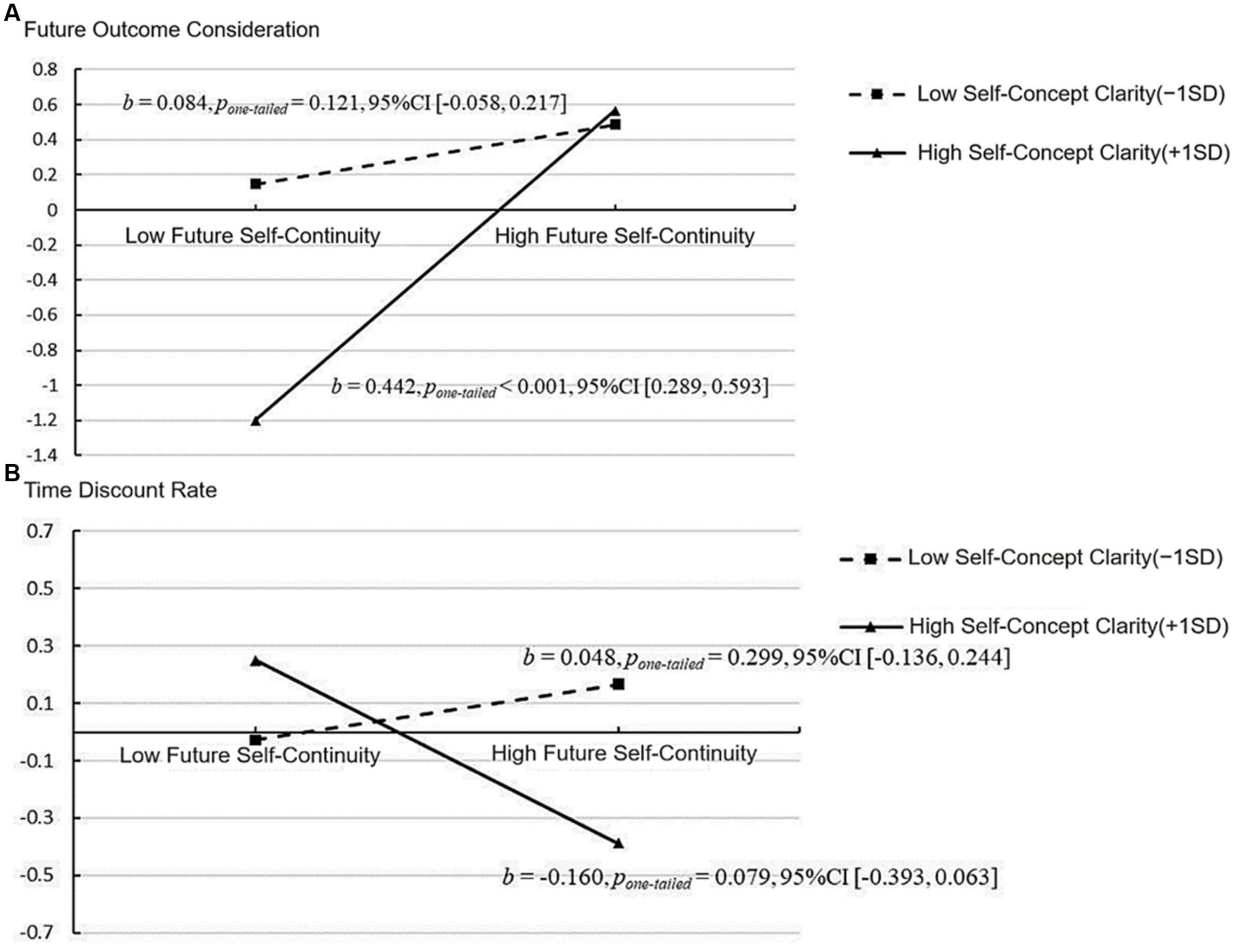
Simple effect analysis results of the mediated regulatory model in Study 2. **(A)** Mediation pathway: Future self-continuity → Future outcome consideration. **(B)** Mediation path: Future self continuity → Intertemporal decision making.

To further elucidate the regulatory role of self-concept clarity, this study conducted a simple slope analysis, as depicted in Figure 6. Figure 6A illustrated the moderating effect of self-concept clarity on the impact of future self-continuity on considerations of future outcomes. The findings indicated that in individuals with lower levels of self-concept clarity (−1SD), the impact of future self-continuity on considerations of future outcomes was not statistically significant (*b* = 0.084, *p_one-tailed_* = 0.121, 95%CI = [−0.058, 0.217]). In individuals with a high level of self-concept clarity (+1SD), future self-continuity significantly and positively influenced considerations of future outcomes (*b* = 0.442, *p_one-tailed_* < 0.001, 95%CI = [0.289, 0.593]). Figure 6B illustrated the moderating effect of self-concept clarity on the impact of future self-continuity on intertemporal decision-making. The findings indicated that irrespective of the level of self-concept clarity, the impact of future self-continuity on the time discount rate of intertemporal decision-making was not statistically significant (−1SD: *b* = 0.048, *p_one-tailed_* = 0.299, 95%CI = [−0.136, 0.244]; +1SD: *b* = −0.160, *p_one-tailed_* = 0.079, 95%CI = [−0.393, 0.063]).

The mediation analysis revealed that in the relationship between future self-continuity and the discount rate of intertemporal decision-making, self-concept clarity had a moderating effect of −0.202, comprising a direct moderating effect of −0.122, an indirect moderating effect of −0.080, and an indirect moderating effect accounting for 39.60%. In a moderated mediation model, the interaction term (X*W) between future self-continuity and self-concept clarity no longer significantly moderated the time discount rate of intertemporal decision-making (*b* = −0.122, *p_one-tailed_* = 0.089, 95%CI = [−0.302, 0.047]). Thus, future results completely mediated the relationship. In individuals with lower levels of self-concept clarity (−1SD), the indirect effects of future outcome consideration on both future self-continuity and the time discount rate for intertemporal decision-making were not significant (*Effect* = −0.030, *p* = 0.123, 95%CI = [−0.097, 0.021]). Conversely, individuals with a high level of self-concept clarity (+1SD) exhibited a significant indirect effect of future outcome consideration on both future self-continuity and the time discount rate of intertemporal decision-making (*Effect* = −0.161, *p* = 0.003, 95%CI = [−0.290, −0.020]).

### 3.3 Discussion of study 2

The research found that future self-continuity significantly negatively affected the time discount rate in intertemporal decision-making, and that self-concept clarity regulated this impact, consistent with Study 1 results. Secondly, based on the moderation model, future outcome considerations were included, and it was found that self-concept clarity significantly moderated the impact of future self-continuity on future outcome considerations. A moderated model was established to validate Hypothesis 3. Individuals with high levels of self-concept clarity can clearly understand their own tendencies, so future self-continuity significantly predicts future outcomes. In individuals with lower levels of self-concept clarity, their self-concept is chaotic, and they lack a clear understanding of their own pursuits and life outcomes, resulting in no significant relationship between future self-continuity and consideration of future outcomes (Campbell, 1990).

Furthermore, the mediating effect of future outcome consideration suggested that the tendency to consider future outcomes was an explanatory mechanism influencing individual intertemporal decision-making. The moderating effect of self-concept clarity indirectly influenced individual intertemporal decision-making through future outcome consideration, supporting the theory that individuals made intertemporal decisions differently due to their tendency to consider the future (Strathman et al., 1994; Rappange et al., 2010). The current results also found that future outcome considerations completely mediated the moderating effect of self-concept clarity on individual intertemporal decision-making, further indicating that future outcome considerations are an important factor influencing the differences in intertemporal decision-making between individuals. Future outcome considerations explained the moderating effect of self-concept clarity on individual intertemporal decision-making.

## 4 Discussion

### 4.1 The impact of future self-continuity on intertemporal decision-making

Two studies indicated that future self-continuity significantly negatively predicted the time discount rate of intertemporal decision-making. This finding suggested that individuals with higher future self-continuity were more inclined to choose delayed options in intertemporal decision-making, while those with lower future self-continuity were more inclined to choose immediate options. This result was consistent with previous research findings (Hershfield, 2011; Zhang and Aggarwal, 2015; Macrae et al., 2017). The impact of future self-continuity on intertemporal decision-making is mainly manifested in an individual’s preference for short-term and long-term options, thereby affecting the degree of time discount (Hershfield H. et al., 2009). Individuals with high future self-continuity believe in a strong connection between their current and future selves, enhancing their attention and importance to their future selves. This inclination leads them to choose delayed and high-yield options more often (Zhang and Aggarwal, 2015). According to the dual processing system theory of decision-making (Stanovich and West, 2000; Kahneman and Frederick, 2002), humans utilize two systems simultaneously during decision-making and thinking. One is an intuitive and automated heuristic system (referred to as the “hot” system), which heavily relies on intuition and operates at a fast processing speed. The other is a rational and conscious analytical system (known as the “cold” system), which heavily relies on information processing and operates at a slow processing speed (Sun et al., 2007). Research indicates that when individuals contemplate a distant future, both the temporal and psychological distances increase (Hershfield H. et al., 2009; Liu et al., 2018). Consequently, their subjective perception of the significance, vividness, and emotional experience of long-term options gradually diminishes, indicating a weakening of their subjective perception of long-term options. When individuals face current decisions, they tend to utilize “hot” systems, focusing more on immediate experiences and recent consequences. The principal theoretical model of future self-continuity posits that similarity, vividness, and positivity are crucial factors collectively influencing future self-continuity. The inclination toward these three characteristics indicates the level of individual future self-continuity, which subsequently impacts intertemporal decision-making (Klineberg, 1968; Hershfield, 2011; Zhang and Aggarwal, 2015). Individuals characterized by high future self-continuity can shape subjective experiences by enhancing the similarity, vividness, and positivity of long-term options, enabling them to gain a better understanding of the long-term impact of current decisions and the benefits of long-term options. Consequently, individuals with lower levels of future self-continuity are more likely to struggle with comprehending and experiencing their future self accurately, leading to increased reliance on the “hot” system and higher time discount rates.

### 4.2 The moderation effect of self-concept clarity

Two studies indicated that self-concept clarity moderated the relationship between future self-continuity and intertemporal decision-making. Among individuals with high levels of self-clarity, future self-continuity significantly and positively predicted intertemporal decision-making. However, among those with lower levels of self-clarity, there was no significant relationship between future self-continuity and intertemporal decision-making, which is consistent with existing research findings (Thaler and Shefrin, 1981; Hershfield, 2011; Jiang et al., 2023). Individuals with higher self-concept clarity can more effectively clarify their goals, values, and priorities, making it easier for them to make decisions that align with their self-concept. This focus on the future leads them to be more committed to achieving long-term goals. Conversely, individuals with lower self-concept clarity are more susceptible to the influence of external information and may consequently become confused about their goals (Campbell et al., 1996). Therefore, individuals with higher self-concept clarity were more likely to perceive the connection between the present and the future, have a clearer understanding of who they are, and understand their important life goals. Individuals with high self-concept clarity were better able to imagine future states and situations, a capability that requires a clear and coherent self-concept. The clearer an individual’s self-concept in different time dimensions, the stronger their self-continuity, leading them to believe that their past, present, and future selves are the same person (Liu et al., 2018). Research showed that when individuals perceived their future selves as realistic and vivid, and viewed them positively, they were more inclined to make sacrifices in the present. These sacrifices might benefit them in the future (Hershfield, 2011). Self-concept plays a regulatory role in intertemporal decision-making, with delayed gratification being a rational choice based on self-regulation (Markus and Nurius, 1986). A high level of self-concept clarity helps individuals guide their behavior and is associated with long-term effort. This clarity helps individuals adjust their current emotions and behaviors in a timely manner, and suppress actions that do not align with their goals (Setterlund and Niedenthal, 1993). Therefore, for individuals with high levels of self-clarity, higher future self-continuity enables them to clearly recognize current and future needs, making it easier to choose delayed and more profitable intertemporal decisions. Conversely, individuals with low self-clarity may lack a clear understanding of their needs and be unaware of the differences between the present and future, resulting in an unclear relationship between their future self-continuity and intertemporal decision-making.

### 4.3 The mediating role of future outcome considerations

The research findings indicated that future outcome considerations mediated the relationship between future self-continuity and intertemporal decision-making. Specifically, individuals with high future self-continuity placed greater emphasis on the impact of current behavior on the future and were more willing to consider the long-term benefits of delayed choices. Conversely, individuals with low future self-continuity tended to consider future outcomes more vaguely and focused more on the satisfaction brought by current choices, which was consistent with previous research findings (Strathman et al., 1994). Future outcome considerations significantly negatively predict the time discount rate in intertemporal decision-making (Rappange et al., 2010). Read et al. (2005) found that individuals who described their future selves were more willing to choose delayed gratification compared to those who described their current selves. Individuals who consider low future outcomes tend to focus more on immediate needs rather than future needs. In contrast, individuals who consider high future outcomes will consider the impact of their behavior on the future (Pozolotina and Olsen, 2019). A series of studies have shown that individuals with high future outcome considerations are positively correlated with personality traits related to self-control, such as a sense of responsibility, delayed gratification, long-term thinking, and future-oriented behavior (Joireman et al., 2001; Peters et al., 2005). Individuals who consider high-level future outcome considerations are more inclined to discount future rewards at a lower rate, indicating that they value future rewards more and are therefore more willing to wait and make short-term sacrifices for long-term benefits.

The research findings further revealed that future outcome considerations fully mediated the moderating effect of self-concept clarity on the relationship between future self-continuity and intertemporal decision-making. Individuals with high self-concept clarity can enhance their consideration of future outcomes through future self-continuity, thereby influencing the time discount rate in intertemporal decision-making. In contrast, individuals with low self-concept clarity have limited influence on their consideration of future outcomes due to future self-continuity. In other words, individuals with high self-concept clarity enhance their future self-continuity by enhancing their consideration of future outcomes, facilitating a more holistic view of their present and future selves. By emphasizing the importance of the future self and making it more vivid, individuals reduce the psychological distance between their present and future selves, altering how they weigh long-term and short-term outcomes in intertemporal decision-making. Likewise, individuals with low self-concept clarity have minimal influence on their consideration of future outcomes because of future self-continuity, which complicates their ability to contemplate the potential impact of future outcomes, resulting in myopic behavior in intertemporal decision-making. In summary, a clear self-concept helps construct a vivid future self-image, enhances imagination for the future, and facilitates individuals’ ability to foresee the future consequences of their current behavior. Therefore, they are more inclined toward future options in intertemporal decision-making.

### 4.4 Limitations and directions for future research

This study explored the moderating effect of self-concept clarity on the influence of future self-continuity on intertemporal decision-making and the mediating effect of future outcome consideration. Using a combination of questionnaires and experiments, this study conducted a comprehensive and in-depth exploration of the factors influencing intertemporal decision-making, enhancing the diversity and applicability of the research methods. However, this study had certain limitations. Firstly, the main focus was on the rate of choosing immediate options. Participants were instructed to make decisions based on their genuine feelings without time constraints at the start of the experiment. Individual differences in response times during the experimental design also affected data analysis. Future research should carefully consider the impact of response times on the conclusions. Secondly, this study employed a decision-making paradigm focused on the monetary domain. Future research should further explore preferences for intertemporal decision-making in non-monetary domains to fully understand the diversity of decision-making behaviors.

## 5 Conclusion

Intertemporal decision-making refers to the process of making choices between current and future outcomes, whether choosing to indulge in immediate happiness or deciding to save money for retirement; both are manifestations of intertemporal decision-making in people’s lives (Peters and Büchel, 2011; Reeck et al., 2017). This study explored the impact of future self-continuity on intertemporal decision-making through two studies, examining the roles of self-concept clarity and consideration of future outcomes. Firstly, the results of this study found that future self-continuity significantly negatively affected the time discount rate of intertemporal decision-making. Secondly, self-concept clarity significantly moderated the impact of future self-continuity on intertemporal decision-making. For individuals with high levels of self-clarity, future self-continuity significantly positively predicted intertemporal decision-making; for individuals with lower levels of self-clarity, there was no significant relationship between future self-continuity and intertemporal decision-making. Finally, future outcome consideration mediated the moderating effect of self-concept clarity on future self-continuity and intertemporal decision-making. Individuals with high self-concept clarity enhanced their future outcome consideration through future self-continuity, affecting the time discount rate in intertemporal decision-making. Individuals with low self-concept clarity were less influenced by future self-continuity in their consideration of future outcomes.

## Data availability statement

The original contributions presented in the study are included in the article/Supplementary material, further inquiries can be directed to the corresponding authors.

## Ethics statement

The studies involving humans were approved by the local Ethics Committee of Hunan Normal University. The studies were conducted in accordance with the local legislation and institutional requirements. The participants provided their written informed consent to participate in this study.

## Author contributions

YY: Validation, Writing – original draft, Writing – review & editing, Visualization. LZ: Data curation, Resources, Writing – original draft. WQ: Conceptualization, Funding acquisition, Supervision, Writing – review & editing. WF: Conceptualization, Methodology, Supervision, Writing – review & editing.

## Footnotes

^1^https://www.wenjuan.com
