# Supplementary material for: The effect of future self-continuity on intertemporal decision making: a mediated moderating model
Source: Front Psychol. 2024 Aug 8;15:1437065. doi: 10.3389/fpsyg.2024.1437065 (PMC11339553; doi:10.3389/fpsyg.2024.1437065)
Supplement: Supplementary file 1 [file Data_Sheet_1.docx]

**The Effect of Future Self-continuity on** **Intertemporal Decision Making:** **a** **Mediated** **Moderating Model——Supplementary materials**

***The monetary-choice questionnaire.***

Imagine that you have completed a piece of work. There are now two ways to receive payment: to receive a smaller amount immediately (Option A); or receive a larger payment at a future date (Option B). There are 27 scenarios in total. Please make a choice in each scenario according to your preference and mark the corresponding option with “√”. Please note that each situation is independent of each other, and there is no right or wrong answer; it is entirely based on personal preference.

| Order | Smaller, Immediate Reward (Option A) | larger, Delayed Reward (Option B) | Delays are in days |
| --- | --- | --- | --- |
| 1 | CNY 238 | CNY 245 | 186 |
| 2 | CNY 378 | CNY 385 | 117 |
| 3 | CNY 546 | CNY 560 | 162 |
| 4 | CNY 196 | CNY 210 | 179 |
| 5 | CNY 329 | CNY 350 | 160 |
| 6 | CNY 560 | CNY 595 | 157 |
| 7 | CNY 154 | CNY 175 | 136 |
| 8 | CNY 378 | CNY 420 | 111 |
| 9 | CNY 469 | CNY 525 | 119 |
| 10 | CNY 175 | CNY 210 | 80 |
| 11 | CNY 343 | CNY 420 | 89 |
| 12 | CNY 483 | CNY 595 | 91 |
| 13 | CNY 133 | CNY 175 | 53 |
| 14 | CNY 280 | CNY 385 | 62 |
| 15 | CNY 385 | CNY 525 | 61 |
| 16 | CNY 168 | CNY 245 | 29 |
| 17 | CNY 238 | CNY 350 | 30 |
| 18 | CNY 378 | CNY 560 | 30 |
| 19 | CNY 98 | CNY 175 | 19 |
| 20 | CNY 189 | CNY 350 | 21 |
| 21 | CNY 287 | CNY 525 | 20 |
| 22 | CNY 105 | CNY 245 | 13 |
| 23 | CNY 175 | CNY 420 | 14 |
| 24 | CNY 231 | CNY 560 | 14 |
| 25 | CNY 77 | CNY 210 | 7 |
| 26 | CNY 140 | CNY 385 | 7 |
| 27 | CNY 217 | CNY 595 | 7 |
